# Genomic sequencing, genome-scale metabolic network reconstruction, and in silico flux analysis of the grape endophytic fungus *Alternaria* sp. MG1

**DOI:** 10.1186/s12934-019-1063-7

**Published:** 2019-01-24

**Authors:** Yao Lu, Chao Ye, Jinxin Che, Xiaoguang Xu, Dongyan Shao, Chunmei Jiang, Yanlin Liu, Junling Shi

**Affiliations:** 1 Key Laboratory for Space Bioscience and Biotechnology, School of Life Sciences, Northwestern Polytechnical University, 127 Youyi West Road, Xi’an, 710072 Shaanxi China; 2 State Key Laboratory of Food Science and Technology, Jiangnan University, 1800 Lihu Road, Wuxi, 214122 Jiangsu China; 3 Department of Biological and Food Engineering, College of Chemical Engineering, Xiangtan University, Xiangtan, 411105 Hunan China; 4 College of Enology, Northwest A&F University, 22 Xinong Road, Yangling, 712100 Shaanxi China

**Keywords:** *Alternaria* sp. MG1, Secondary metabolites, Genome-scale metabolic model, Resveratrol, Constraints-based flux analysis

## Abstract

**Background:**

*Alternaria* sp. MG1, an endophytic fungus isolated from grape, is a native producer of resveratrol, which has important application potential. However, the metabolic characteristics and physiological behavior of MG1 still remains mostly unraveled. In addition, the resveratrol production of the strain is low. Thus, the whole-genome sequencing is highly required for elucidating the resveratrol biosynthesis pathway. Furthermore, the metabolic network model of MG1 was constructed to provide a computational guided approach for improving the yield of resveratrol.

**Results:**

Firstly, a draft genomic sequence of MG1 was generated with a size of 34.7 Mbp and a GC content of 50.96%. Genome annotation indicated that MG1 possessed complete biosynthesis pathways for stilbenoids, flavonoids, and lignins. Eight secondary metabolites involved in these pathways were detected by GC–MS analysis, confirming the metabolic diversity of MG1. Furthermore, the first genome-scale metabolic network of *Alternaria* sp. MG1 (named *i*YL1539) was reconstructed, accounting for 1539 genes, 2231 metabolites, and 2255 reactions. The model was validated qualitatively and quantitatively by comparing the in silico simulation with experimental data, and the results showed a high consistency. In *i*YL1539, 56 genes were identified as growth essential in rich medium. According to constraint-based analysis, the importance of cofactors for the resveratrol biosynthesis was successfully demonstrated. Ethanol addition was predicted in silico to be an effective method to improve resveratrol production by strengthening acetyl-CoA synthesis and pentose phosphate pathway, and was verified experimentally with a 26.31% increase of resveratrol. Finally, 6 genes were identified as potential targets for resveratrol over-production by the recently developed methodology. The target-genes were validated using salicylic acid as elicitor, leading to an increase of resveratrol yield by 33.32% and the expression of gene *4CL* and *CHS* by 1.8- and 1.6-fold, respectively.

**Conclusions:**

This study details the diverse capability and key genes of *Alternaria* sp. MG1 to produce multiple secondary metabolites. The first model of the species *Alternaria* was constructed, providing an overall understanding of the physiological behavior and metabolic characteristics of MG1. The model is a highly useful tool for enhancing productivity by rational design of the metabolic pathway for resveratrol and other secondary metabolites.

**Electronic supplementary material:**

The online version of this article (10.1186/s12934-019-1063-7) contains supplementary material, which is available to authorized users.

## Background

Endophytic fungi from plants have attracted increasing scientific attention due to their capability to produce high value bioactive compounds [1]. During the evolutionary process of plant symbiosis, many endophytic fungi obtained the capability to synthesize secondary metabolites similar to their host plants [2, 3]. Under the current market demand, endophytic fungi exhibit significant potential for producing plant-original drugs and functional compounds, such as taxol, which is a renowned antitumor agent that was originally isolated from the bark of the Pacific Yew, *Taxus brevifolia* [4]. Moreover, endophytes offer the advantages of accumulating higher concentrations of functional compounds that would be toxic to genetically modified *Escherichia coli* and yeast since they possess higher resistance to their self-produced metabolites [5]. *Alternaria* sp. MG1 is an endophytic fungus previously isolated from the cob of *Vitis vinifera* L. cv. Merlot that could stably produce resveratrol. Resveratrol is a stilbene with multiple functions including antitumor, cardioprotective, antioxidant, lifespan-extending, and anti-inflammatory activities [6, 7]. Compared to the complex procedures and toxic organic solvents needed for its chemical synthesis [8, 9], genomic instability for plant cell culture [10], and the time-consumption and product inhibition for genetically modified *E. coli* and yeast [11], *Alternaria* sp. MG1 showed notable advantages: It does not require genetic modification and offers stable production of resveratrol in the microbial fermentation in vitro. However, the production of resveratrol by *Alternaria* sp. MG1 was low to be directly used as an industrial resveratrol producer. It is therefore necessary to obtain a full understanding of the resveratrol biosynthesis pathway in *Alternaria* sp. MG1, since it has not been verified in any microorganisms at the gene level.

Genome-scale metabolic modeling (GSMM) is a novel systematic biology technology for the construction of a framework for the integrative analysis of the metabolic functions of a microorganism. GSMM is conducted based on genome annotation, ‘omics’ data sets, and legacy knowledge [12], clarifying the relationships between genes, proteins, and reactions. GSMM results provide important support that guides metabolic engineering and strain improvement, integrate high-throughput data by providing a visualization platform for the analysis of multi-omic information, and investigate strain evolution [13, 14]. So far, more than 300 GSMMs, covering approximately 150 microbial species, have been successfully established [15]. For example, GSMM analysis indicated the central role of secondary replicons for the growth of the legume symbiont *Sinorhizobium meliloti* in three ecological niches and clarified the specialized function of these replicons for host-associated niche adaption [16]. The model *i*HZ771 of *Komagataeibacter nataicola* RZS01 was successfully constructed and used to predict potential targets for the over-production of cellulose in silico [17]. Based on GSMM, it is easy to obtain a comprehensive understanding of the physiological behavior and metabolic characteristics of a microorganism under different environmental or genetic disturbance. However, there is no report on the GSMM of *Alternaria* sp. up to now.

In this study, the whole genome of *Alternaria* sp. MG1 was de novo sequenced, assembled, annotated, and subsequently used for the reconstruction of a GSMM of *Alternaria* sp. strain MG1, which offers a systematic insight into the metabolic characteristics involved in the production of resveratrol. The resveratrol biosynthesis pathway was verified by flux analysis in the model. The roles of cofactors in the regulation of resveratrol biosynthesis were investigated, and both biochemical and genetic strategies were proposed for the improvement of resveratrol production. Additionally, GC/HPLC–MS was conducted to mine the capability of metabolic diversity of MG1. The results reported herein provide a reliable platform and reference for future studies on the resveratrol biosynthesis pathway in microorganisms and are a pre-requisite for the exploitation of other valuable secondary metabolites produced by *Alternaria* sp. MG1.

## Results and discussion

### Genome sequencing and characteristics

To obtain a comprehensive understanding of the functional genes, especially those response for the biosynthesis of resveratrol and other functional metabolites in *Alternaria* sp. MG1, genome sequencing and characterization were conducted on this strain. The 34.7 Mb genome sequence with a GC content of 50.96% was acquired from a 600 bp pair-end library and a 3 kb pair-end library. The Q20 values of the short-insert and long-insert libraries were 91.98% and 97.45%, respectively. 132 scaffolds (> 1 kb) and 1506 contigs were assembled with N50 sizes of 1,661,510 and 44,208 bp, respectively. The repeat sequences number was 1771 and the length was 115,952 bp. A total of 13,606 genes were predicted, and 37 rRNA and 129 tRNA were identified. 96.19% out of 13,606 predicted protein-coding genes were successfully functionally annotated via aligning of the sequence with a variety of databases as listed in Table 1.

**Table 1:** Genome characteristics of *Alternaria* sp. MG1

General features	
Genome size (Mb)	34.7
GC content (%)	50.96
Number of scaffolds	132
Scaffold N50 (bp)	1,661,510
Number of contigs	1506
Contig N50 (bp)	44,208
Number of protein-coding sequences	13,606
Properties of gene annotation	
Number of rRNA genes	37
Number of tRNA genes	129
COG annotation	5037
GO annotation	6602
KAAS annotation	2663
KOG annotation	6413
Pfam annotation	8955
Swissprot annotation	8330
TrEMBL annotation	13,055
Nr annotation	13,045
Nt annotation	10,080
Total	13,088

### Annotation of the biosynthesis pathways of secondary metabolites including resveratrol

**Fig. 1:**
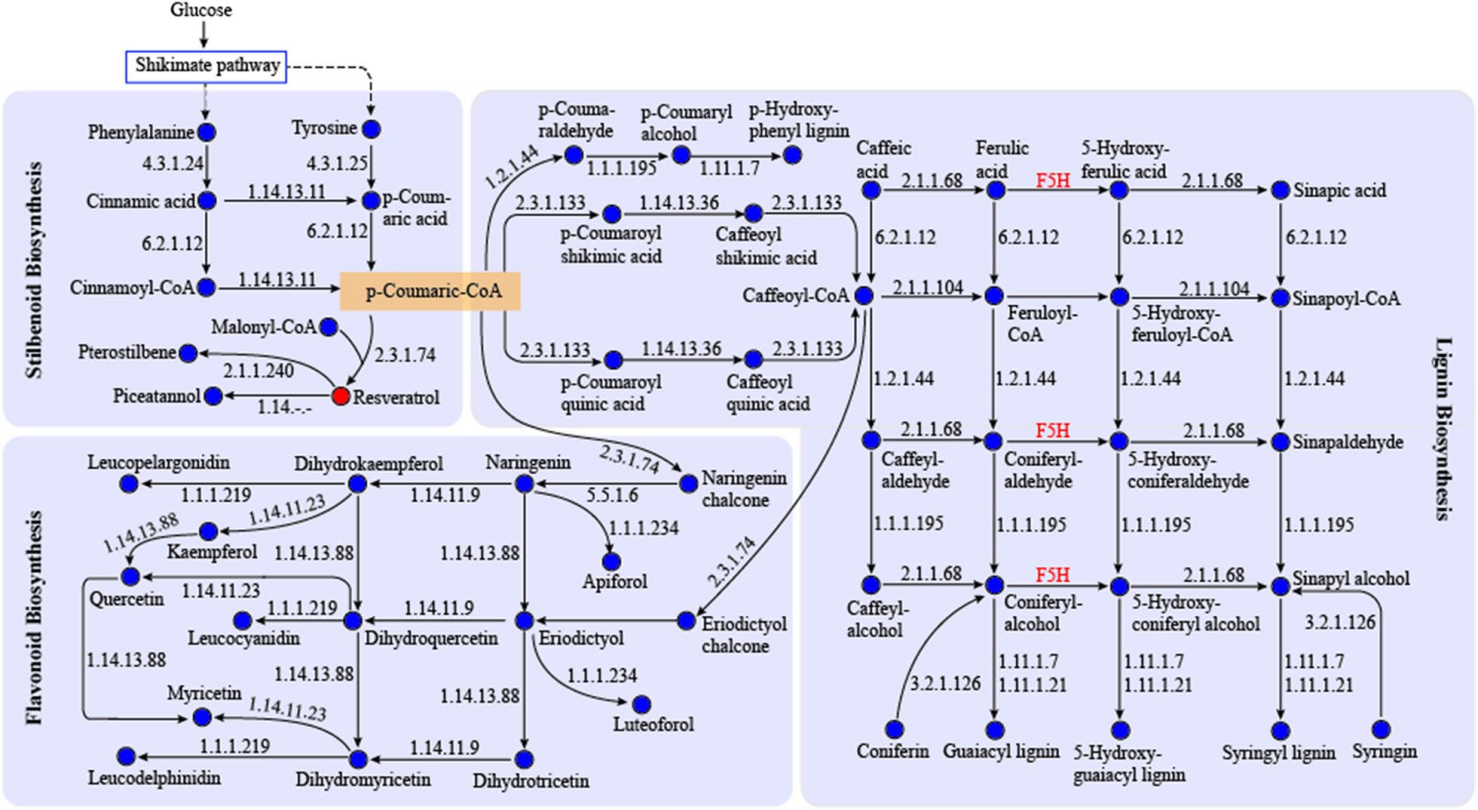
Phenylpropanoid and downstream secondary metabolites synthesis pathways in *Alternaria* sp. MG1. *F5H* ferulate-5-hydroxylase. Enzymes in red font are present in *Vitis vinifera* but are not annotated in *Alternaria* sp. MG1. Dashed arrows indicate the phenylalanine/tyrosine synthesis pathways that are not listed in detail

Gene annotation was conducted based on the genome sequencing results. This provided an overall information on the functional genes that are responsible for the biosynthesis of different metabolites. As expected, 21 proteins encoded by more than one gene each, were identified in the phenylpropanoid pathways, especially those responsible for the biosynthesis of stilbenes, flavonoids, and lignins, indicating the presence of a resveratrol biosynthesis pathway and the metabolic diversity in the strain (Fig. 1). For the synthesis of resveratrol, the key genes of the upstream metabolic pathway 4-coumarate coenzyme A ligase (4CL, 6.2.1.12) and cinnamate 4-hydroxylase (C4H, 1.14.13.11) were identified. Stilbene synthase (STS, 2.3.1.95), which catalyzes the last step in resveratrol synthesis, was not annotated in any database, while chalcone synthase (CHS, 2.3.1.74) was identified. Several of the *CHS*-like genes could exhibit activities in catalyzing stilbene formation. For example, the *CHS* gene cloned from *Psilotum nudum* showed pinosylvin synthase activity and that from *Hydrangea macrophylla* showed stilbenecarboxylate synthase activity to catalyze the formation of 5-hydroxy-lunularic acid [18]. In addition, STSs and CHSs showed high similarity at the amino acid level, which often confuses the distinction between both enzymes in gene annotation [19]. For instance, when three initially annotated *STS* genes (*Morus atropurpurea* Roxb.) were co-expressed with Ma*4CL*, the formation of naringenin, not resveratrol, was detected [20]. Furthermore, although CHS is naturally responsible for the naringenin biosynthesis, cross reactivity between CHS and STS has been demonstrated with CHS forming resveratrol and STS forming naringenin [21]. In the presence of *p*-coumaroyl-CoA as the substrate, STS isolated from *Rheum tataricum* L. showed in vitro activity to form resveratrol as well as trace amounts of naringenin chalcone [22]. All these results indicated that CHS and STS had overlapped functions for the formation of flavonoids and stilbene. Therefore, it was reasonable to assume that the identified CHS in our study possessed activity to catalyze the formation of resveratrol. Very recently, Wenderoth et al. [23] established a CRISPR/Cas9 system and successfully inactivated two genes of the melanin-biosynthesis pathway in *Alternaria alternata*. This indicated a possible method to verify the capability of the function of identified chalcone synthase in biosynthesizing resveratrol. Phenylalanine/tyrosine ammonia-lyase (PTAL, 4.3.1.25) in the resveratrol biosynthesis pathway was also successfully identified by BLASTp through further mining the transcriptome data. This is consistent with previous findings that indicated the *Alternaria* sp. MG1 could produce resveratrol using tyrosine as the sole substrate [24]. These results enriched the resveratrol synthesis pathway of the *Alternaria* sp. MG1 at the gene level.

The enzymes catalyzing resveratrol into pterostilbene and piceatannol were also successfully annotated as trans-resveratrol di-*O*-methyltransferase (ROMT, 2.1.1.240) and piceatannol synthase in *Alternaria* sp. MG1, respectively. Pterostilbene is a dimethyl ether analog of resveratrol that exhibits multiple properties e.g., cancer chemopreventive, anti-oxidative, antifungal, and hypolipidemic properties [25]. The accumulation of pterostilbene in MG1 was identified using UPLC-Qtof-MS (Additional file 1: Figure S1), since there is no standard of this compound in the used database of GC–MS. Piceatannol is a resveratrol analogue that has antitumorigenic and immunosuppressive activities, as well as stronger antioxidant activity than resveratrol. It has been suggested to have potential for the development as an antiarrhythmic agent [26]. The specific enzyme responsible for synthesizing piceatannol has been assigned an obscure EC number (1.14.-.-) that belongs to the cytochrome P450 family. CYP1B1 (the cytochrome P450 enzyme) has been reported to have capability for the conversion of resveratrol into piceatannol [26]. Piceatannol accumulation was also detected in the culture of *Alternaria* sp. MG1 using gas chromatography–mass spectrometry (Table 2).

**Table 2:** Stilbenoid, lignin, and flavonoid compounds detected in *Alternaria* sp. MG1

Classification	CAS	Putative identification	RI	RT	Biological activity	References
Stilbenoid	501-36-0	Resveratrol	843,980	22.5333	Anticancer, antidiabetic, cardioprotective, antioxidant, neuroprotective, lifespan-extending	[7, 78, 79]
	10083-24-6	Piceatannol	985,592	25.9867	Antioxidant, anticancer, chemopreventive, immunomodulatory, anti-adipogenesis, anti-proliferative and anti-inflammatory	[27, 80]
Lignin	331-39-5	Caffeic acid	749,379	21.9350	Antioxidant, anti-inflammatory, anti-tumor, antiurolithic, antithrombosis, antihypertensive, antiviral	[28, 81, 82]
	537-33-7	Sinapyl alcohol	682,370	18.5742	Anti-inflammatory, antinociceptive	[29]
	458-35-5	Coniferyl alcohol	675,750	20.1625	Antibacterial	[30]
	530-59-6	*cis*-Sinapinic acid	716,675	21.1475	Anxiolytic, anti-inflammatory, antioxidant, antihyperglycemic, antihypertensive, cardiovascular protective	[31, 83]
Flavonoid	93602-28-9	Naringenin	1,017,864	28.3933	Anti-inflammatory, antioxidant, anti-microbial, antiviral, anti-amnestic, anti-thrombotic, anti-tumorigenic, anti-atherosclerotic, and anti-hypercholesterolemic	[32, 84]
	480-18-2	Taxifolin	1,007,499	26.5208	Antioxidant, anti-inflammatory, antitumor, cardioprotective, hepatoprotective	[33, 85]

*CAS* chemical abstracts service (CAS) registry number, *RI* retention index, *RT* retention time

In addition, shikimate *O*-hydroxycinnamoyltransferase (HCT, 2.3.1.133), the enzyme responsible for driving metabolic flux from *p*-coumaric CoA to the lignin biosynthesis pathway was successfully annotated in *Alternaria* sp. MG1. However, ferulate-5-hydroxylase (F5H), which is a key gene in the lignin biosynthesis pathway (in *Vitis vinifera*), was not found in *Alternaria* sp. MG1. Moreover, key enzymes in the flavonoid biosynthesis pathway from *p*-coumaric CoA to naringenin chalcone were also annotated.

### Multiple phenylpropanoid biosynthesis pathways identified in *Alternaria* sp. MG1

Phenylpropanoid biosynthesis has the same precursors and key genes in the upstream pathway for downstream biosynthesis of stilbenes, flavonoids, and lignins. Therefore, the functional genes in these pathways were also annotated based on the genomic data of *Alternaria* sp. MG1, and the corresponding metabolites were detected via GC–MS analysis. As a result, a total of 379 compounds were detected inside cells and in the cell-free culture by setting the identification standard of similarity value greater than 200 (Additional file 2). Two stilbenes (resveratrol and piceatannol), two flavonoids (naringenin and taxifolin), and four lignins (caffeic acid, sinapyl alcohol, coniferyl alcohol, and *cis*-sinapinic acid) were identified as products of *Alternaria* sp. MG1 (Table 2). These compounds have been found to possess multifunctional functions e.g., anticancer, anti-inflammatory, antioxidant, and anti-microbial activities [27–33]. Several of them are also important precursors for drug processing, e.g., coniferyl alcohol is the precursor for the synthesis of ferulic acid and pinoresinol [34, 35]. In the field of environmental protection, coniferyl alcohol also showed significant potential as a marker compound for wood smoke emissions in the atmosphere [36]. More metabolites would be detected if there were more standards in the used database. Overall, the obtained results indicated that *Alternaria* sp. MG1 possessed multiple metabolic pathways and provided diverse gene resources for the production of functional compounds, especially those normally produced by plants at low levels.

### Construction of the genome-scale metabolic model of *Alternaria* sp. MG1

Construction of GSMM is an efficient method toward understanding the overall metabolic network and predicting the potential application of microorganisms for producing valuable metabolites. Taking the phenylpropanoid-resveratrol biosynthesis pathway as an example, we constructed GSMM of *Alternaria* sp. MG1 based on genome annotation. The draft model was obtained via homologous alignment with reference strains using previously described procedures, followed by manual curation. Firstly, 97 reactions in the biosynthesis pathways of phenylpropanoid, stilbene synthesis, flavonoid synthesis, and lignin biosynthesis were added to the model by referring to the KEGG database, BLASTp, and literature reports [24, 37]. A total of 220 transport reactions annotated from the TCDB database and 133 exchange reactions were also added into the model *i*YL1539. Secondly, biomass equation was built and used as objective function to simulate in silico flux values. The biomass comprised 32% cell wall, 20% proteins, 15% mannitol, 15% ash, 14% lipid, and 4% nucleic acid (Additional file 3). Thirdly, a total of 149 redundant and repeated reactions were removed from the model after careful checking of the reaction reversibility and both mass and charge balances. Finally, 43 reaction filling gaps were added to complete the metabolic pathways. For example, the model could not utilize rhamnose as the sole carbon source to maintain cell growth, which contrasted with published literature [38]. Therefore, rhamnose transport and l-rhamnose: NADP+ 1-oxidoreductase reactions were added to the model. Additionally, spontaneous reactions as well as sink and demand reactions were also added to the model to improve both connectivity and coverage of the metabolic network. After an assortment of manual refinements, the complete GSMM encompassing 2255 reactions, 2231 metabolites, and 1539 genes (11.31% of the total protein-coding genes) was constructed and named *i*YL1539 (Additional file 4).

In the model *i*YL1539, eight compartments (cytoplasmic, extracellular, mitochondrial, nuclear, plasma membrane, peroxisome, endoplasmic reticulum, and vacuole) were linked via 140 trans-plasma membrane, 75 cytoplasmic-mitochondrial, three cytoplasmic-nuclear, one cytoplasmic-peroxisome, one cytoplasmic-vacuole transport reactions, and 133 exchange reactions (Additional file 4). Among these, 54.84% protein-coding genes linking 1105 reactions were situated in the cytosol (the main representative), followed by mitochondria (19.62%; 423 reactions). It should be noted that the vast majority of enzymes responsible for phenylpropanoid and resveratrol biosynthesis pathways were localized in the cytoplasm. Therefore, it can be deduced that resveratrol might be biosynthesized in the cytoplasm and subsequently secreted to the extracellular space. The secretion of resveratrol from *V. vinifera* cells to the growth medium has been found and the active transport mechanism has been demonstrated to involve ATP-binding cassette (ABC) transporters, or H^+^-gradient-dependent mechanism via H^+^-antiport [39]. Additionally, Vos et al. [40] successfully overexpressed the *SNQ2* gene, which encodes an ABC transporter to optimize the resveratrol export of engineered *Saccharomyces cerevisiae*. Interestingly, 23 ABC transporters were successfully identified in *Alternaria* sp. MG1 (Additional file 1: Table S1), 21 of which localized in the plasma membrane. These ABC transporters might be responsible for resveratrol secretion.

### Identification of essential genes in iYL1539 for cell growth

**Fig. 2:**
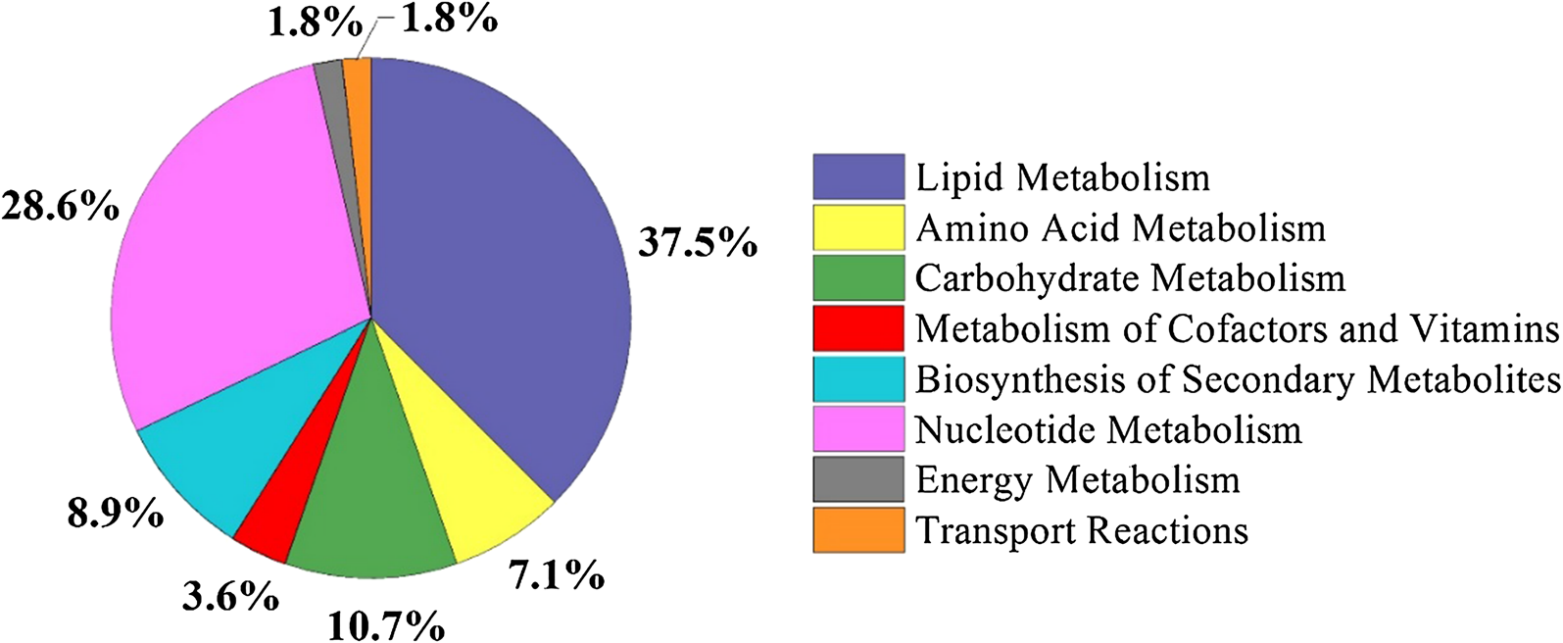
Distribution of essential genes involved in metabolic subsystems

Similar to previous studies, potato-dextrose broth medium (PDB) was used as the medium to cultivate *Alternaria* sp. MG1 for the identification of essential genes for cell growth, because the strain showed very poor growth in synthetic media. PDB is rich in carbohydrates, proteins, and other nutrients. Here, the maximum uptake rates of glucose and 20 amino acids were set to 2.0, and 0.01 mmol/gDW/h, respectively, based on minimal medium [41]. As a result, 56 genes were identified as essential genes for cell growth (Additional file 5). More than 60% of these genes were involved in the lipid (37.5%) and nucleic acid metabolism (28.6%), followed by the carbohydrate metabolism (10.7%, Fig. 2). Furthermore, 8.9% of the essential genes were pivotal for the synthesis of secondary metabolites. For instance, Gglean003194.1 was identified as an essential gene encoding hydroxymethylglutaryl-CoA reductase, catalyzing the formation of mevalonate, which is an intermediate related to terpenoid backbone biosynthesis. Generally, fewer essential genes are required for cell growth on complex medium than on synthetic medium [42]. The essentialities of the identified genes were also validated by DEG database [41] and 52 genes were found to be essential in sharing high homology with reference organisms (Additional file 5). Of the 56 essential genes obtained from cells cultivated in PDB medium, 52 genes (92.9%) matched with DEG database and the other four genes that did not match were mainly involved in the lipid metabolism. This implied that the lipid metabolism would play a vital role in the cellular growth of MG1. In order to verify this assumption, cerulenin, a specific inhibitor of fatty acid biosynthesis, was used to investigate its effect on the growth of MG1. The results indicated that cerulenin treatment significantly inhibited the cellular growth of MG1 with much smaller colony than control (Additional file 1: Figure S2). This demonstrated that lipid metabolism was essential to the growth of MG1. More important, the analysis of essential genes should be reliable because similar results were obtained with two different methods.

### Validation of model iYL1539

#### Model verification via utilization of carbon and nitrogen sources

To assess the practicability of model *i*YL1539, the growth phenotypes of *Alternaria* sp. MG1 were validated by comparing the predicted results in silico with those reported in relevant publications. Overall, the agreement between the simulated results and previous published studies achieved 85% (17/20) and 92% (11/12) for all tested carbon and nitrogen sources, respectively (Table 3). Discrepancies, where the strain showed the ability to metabolize, while the model cannot be used to predict biomass production, were mainly due to the presence of gene annotation gaps, missing transport reactions, and unknown synthetic pathways of the specific substrate [16]. For instance, pectin or citrate could not be used as sole carbon sources to maintain cell growth in the model due to incomplete genomic annotation. The model could not utilize acetamide because the metabolism of acetamide in *Alternaria* sp. has not been reported until now. These inconsistencies required the improvement of the model accuracy by purposefully checking and filling metabolic gaps. For example, after adding the reaction converting dextrin to glucose by dextrin 6-glucanohydrolase (EC: 3.2.1.10, encode by Gglean004959.1) and the corresponding transport reaction of dextrin, the model showed the capability to predict the cell growth using dextrin as carbon source.

**Table 3:** Qualitative and quantitative validation of *i*YL1539

Substrates	In vivo	In silico	References
Prediction of growth phenotypes of *Alternaria* sp. MG1 on different carbon and nitrogen sources			
Carbon sources			
Glucose	+	+	[24]
Fructose	+	+	[24]
Galactose	+	+	[86]
Lactose	+	+	[24]
Maltose	+	+	[86]
Mannose	+	+	[86]
Pectin	+	−	[86]
Ribose	+	+	[86]
Starch*	+	+	[24]
Sucrose*	+	+	[24]
Xylose	+	+	[86]
Arabinose	+	+	[38]
Rhamnose*	+	+	[38]
Raffinose	+	+	[38]
Glycerol	+	+	[87]
Dextrin*	+	+	[24]
Citrate	+	−	[87]
Polygalacturonic acid	+	−	[88]
Cellobiose	+	+	[89]
Sorbose	+	+	[89]
Nitrogen sources			
NH_4_Cl	+	+	[24]
KNO_3_	+	+	[24]
NaNO_2_	+	+	[86]
Acetamide	+	−	[38]
Urea	+	+	[24]
Glycine	+	+	[89]
Glutamate	+	+	[89]
Arginine	+	+	[89]
Alanine	+	+	[89]
Aspartate	+	+	[89]
Asparagine	+	+	[89]
Tyrosine	+	+	[89]

Comparison of in silico and in vivo growth rates			
Constraints (mmol/gDW/h)		Growth rate (h^−1^)	
Consumption rate	RES synthesis rate	In vivo	In silico
Glc (v = 1.65)	8.36E−6	0.1000	0.1013
Suc (v = 0.73)	1.50E−6	0.0893	0.0885

*Glc* glucose, *Suc* sucrose, *RES* resveratrol

+ for growth/− for non-growth; * represents *Alternaria* sp. MG1 can utilize after filling gaps

#### Quantitative validation of *i*YL1539 for cell growth

**Fig. 3:**
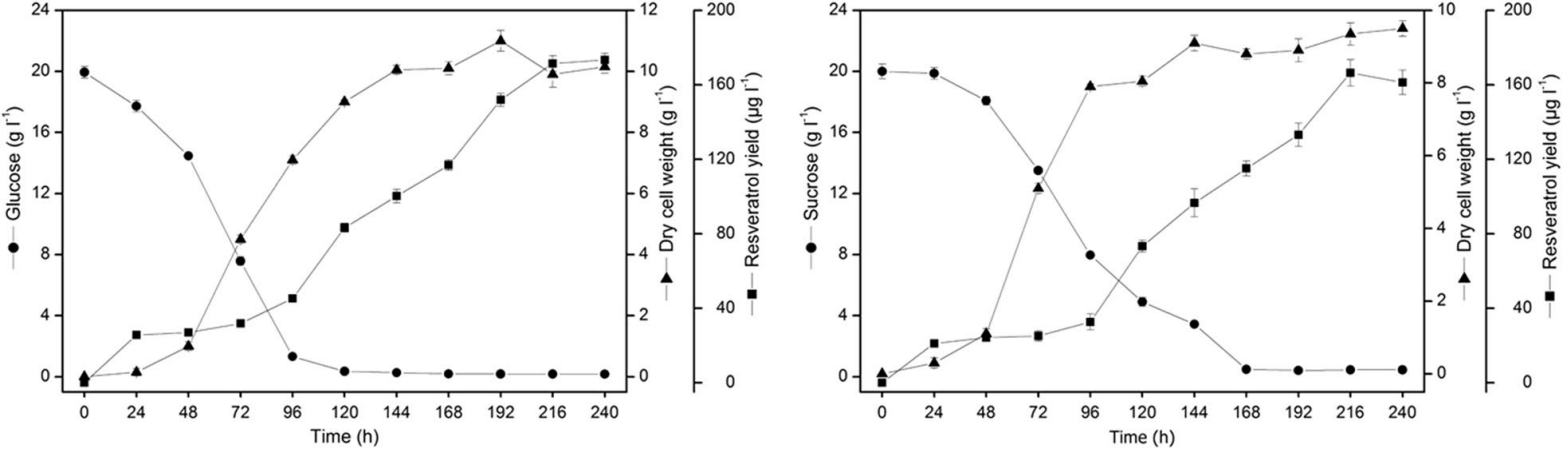
Fermentation diagram of *Alternaria* sp. MG1 for different carbon sources

Validation of the constructed model with experimental data is essential for the evaluation of its practicability. According to a previous study [43], the glucose consumption rate was set to 2.0 mmol/gDW/h, and the maximum uptake rate of all 20 amino acids were set to 0.01 mmol/gDW/h [41] based on minimal medium in the model. In addition, exchange fluxes of Na^+^, K^+^, and Fe^2+^ remained unconstrained to provide basic minerals for cell growth. Under these conditions, the predicted cell growth rate in silico was 0.1247 h^−1^, which was consistent with the literature derived value of 0.1104 h^−1^ (12.95% of the deviation). Still, different physiological characteristics were found among different species. Hence, the fermentation curves (Fig. 3) of *Alternaria* sp. MG1 in PDB medium were investigated to more accurately predict the cell growth capability and resveratrol biosynthesis. As shown in Table 3, the predicted results were matched experimental values with differences of 1.3% and 0.9% when glucose and sucrose were used as carbon sources, respectively. All of these results indicate the high reliability of *i*YL1539 and can therefore be used for further analysis.

### Effect of cofactors on the resveratrol biosynthesis

**Fig. 4:**
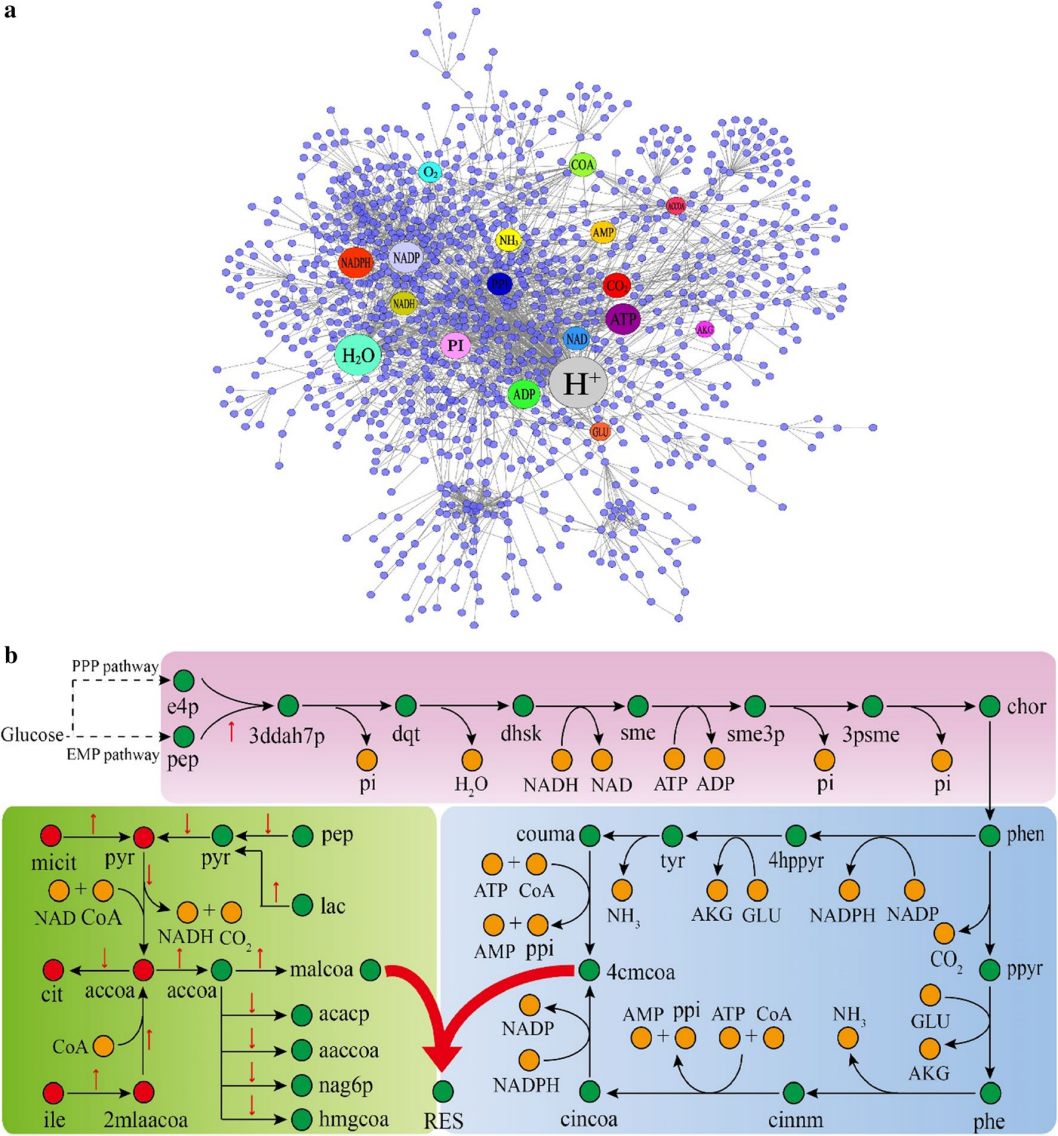
The role of cofactors in the biosynthesis of resveratrol. **a** Metabolic network graph connecting metabolites, genes, and reactions with fluxes during resveratrol biosynthesis. The highlighted circles represent metabolites with high connectivity in the model. *ACCOA* acetyl-CoA, *AKG* 2-oxoglutarate, *GLU* glutamate, *PI* phosphate, *PPI* diphosphate. **b** Cofactors that participated in resveratrol biosynthesis pathway based on GSMM. Three modules represent the shikimate pathway (purple), phenylpropanoid pathway (blue), and the malonyl-CoA synthesis pathway (green), respectively. Dashed arrows indicate the pathways that are not listed in detail. Red arrows indicate the flow changes in response to increasing resveratrol production from 0.0073 to 0.02 mmol/gDW/h. Green/red circles: metabolites in cytosol/mitochondria, orange circles: cofactors. *PPP pathway* pentose phosphate pathway, *EMP pathway* glycolysis pathway, *e4p* d-erythrose 4-phosphate, *pep* phosphoenol pyruvate, *3ddah7p* 2-dehydro-3-deoxy-d-arabino-heptonate 7-phosphate, *dqt* 3-dehydroquinate, *dhsk* 3-dehydroshikimate, *sme* shikimate, *sme3p* shikimate 3-phosphate, *3psme* 5-*O*-(1-carboxyvinyl)-3-phosphoshikimate, *chor* chorismate, *phen* prephenate, *4hppyr* 4-hydroxyphenylpyruvate, *ty* l-tyrosine, *couma* 4-coumarate, *ppyr* phenylpyruvate, *phe* l-phenylalanine, *cinnm* trans-cinnamate, *cincoa* cinnamoyl-CoA, *4cmcoa p*-coumaroyl-CoA, *malcoa* malonyl-CoA, *2mlaacoa* 2-methylacetoacetyl-CoA, *ile* l-isoleucine, *pyr* pyruvate, *micit* methylisocitrate, *lac* (R)-lactate, *cit* citrate, *acacp* acetyl-[acyl-carrier protein], *aaccoa* acetoacetyl-CoA, *nag6p N*-acetyl-d-glucosamine 6-phosphate, *hmgcoa* (*S*)-3-hydroxy-3-methylglutaryl-CoA, *RES* resveratrol

Cofactors play an important role for the biosynthesis of resveratrol at different steps in the whole biosynthesis pathway. Tracing the flow of cofactors in the model could reflect the metabolite connectivity, the total number of reactions that a metabolite involved in the model [44]. Under conditions where the glucose consumption rate was set to 1.0 mmol/gDW/h, growth rate 0.055 h^−1^, and the uptake rates of phenylalanine and tyrosine 1E−06 mmol/gDW/h, flux balance analysis (FBA) was used to investigate the metabolic flux using the resveratrol exchange reaction as the objective function in the model *i*YL1539. As shown in Fig. 4a, 18 metabolites showed high connectivity with reactions including H^+^ (149), H_2_O [77], NADPH [47], NADP [47], ATP [44], ADP [41], phosphate [38], NAD [28], NADH [28], CO_2_ [27], CoA [19], O_2_ [18], AMP [17], glutamate [17], diphosphate [16], NH_3_ [15], 2-oxoglutarate [10], and acetyl-CoA [9] in order. They were mainly related to the metabolism of energy and cofactors. Several of these were directly involved in the resveratrol synthesis, while others were involved in the synthesis of intermediates in the resveratrol biosynthesis pathway or were used to maintain cell growth (Fig. 4b). For instance, glutamate and its precursor 2-oxoglutarate showed high connectivity, indicating that they played more important roles than other amino acids in the resveratrol biosynthesis by *Alternaria* sp. MG1.

Among the cofactors, we extensively analyzed the effect of acetyl-CoA on resveratrol production by *Alternaria* sp. MG1 because it is an essential precursor for the synthesis of malonyl-CoA in the model. Malonyl-CoA and *p*-coumaroyl-CoA are direct precursors for the biosynthesis of resveratrol. In the model *i*YL1539, acetyl-CoA could be generated from 2-methylacetoacetyl-CoA or pyruvate (Fig. 4b). 2-Methylacetoacetyl-CoA was converted from l-isoleucine degradation. Pyruvate was converted from methylisocitrate, phosphoenol pyruvate, or (R)-lactate. Predicted by model *i*YL1539, when the resveratrol production increased from 0.0073 mmol/gDW/h (μ = 0.055 h^−1^) to 0.02 mmol/gDW/h (μ = 0.0519 h^−1^), the mass flux from 2-methylacetoacetyl-CoA to acetyl-CoA increased (1.90%), while that from pyruvate to acetyl-CoA decreased (0.62%) very slightly, despite the increasing conversion from methylisocitrate and lactate to pyruvate (by 4.84%) (Additional file 6). The decrease flux from pyruvate to acetyl-CoA could be explained because another important precursor phosphoenol pyruvate of pyruvate converted more flow from pyruvate synthesis to the shikimate pathway, leading to a decreased flux (1.40%) from phosphoenol pyruvate to pyruvate. For acetyl-CoA consumption, acetyl-CoA could be consumed by the pathways of resveratrol synthesis, fatty acid synthesis, amino sugar metabolism, and terpenoid backbone biosynthesis. FBA analysis showed noticeably decreasing consumption of acetyl-CoA by the pathways other than resveratrol biosynthesis (22.55%) as resveratrol production increased. Specifically, the fatty acid synthesis (acacp and aaccoa) decreased by 11.31% and the conversion from malonyl-CoA to malonyl-ACP decreased by 5.64%. This indicated that fatty acid synthesis was significantly inhibited when the flux of resveratrol biosynthesis increased. Consequently, downregulation of fatty acid synthesis might be an effective strategy to enhance the resveratrol production by *Alternaria* sp. MG1. Blocking the fatty acid synthesis has been developed as an efficient method to enhance the resveratrol production by genetically modified *Escherichia coli* [45].

In addition, it should be mentioned that the decreased consumption of acetyl-CoA in the amino sugar metabolism, such as the biosynthesis of *N*-acetyl-d-glucosamine 6-phosphate (precursor of chitin), influenced cell growth and resulted in a decrease of biomass. This implied that resveratrol biosynthesis competed with cellular growth in mass flux. Therefore, keeping cells under starvation conditions might be helpful for the increase of resveratrol biosynthesis by *Alternaria* sp. MG1, which was consistent with experimental data [46].

The important roles of these cofactors for metabolite synthesis were demonstrated in other studies [47]. The first genome-scale cofactor metabolic model, *i*cmNX6434, has been constructed to elucidate the effects of these cofactors on cell growth, metabolic flux, and industrial robustness [48].

### Regulation of resveratrol biosynthesis based on GSMM

**Fig. 5:**
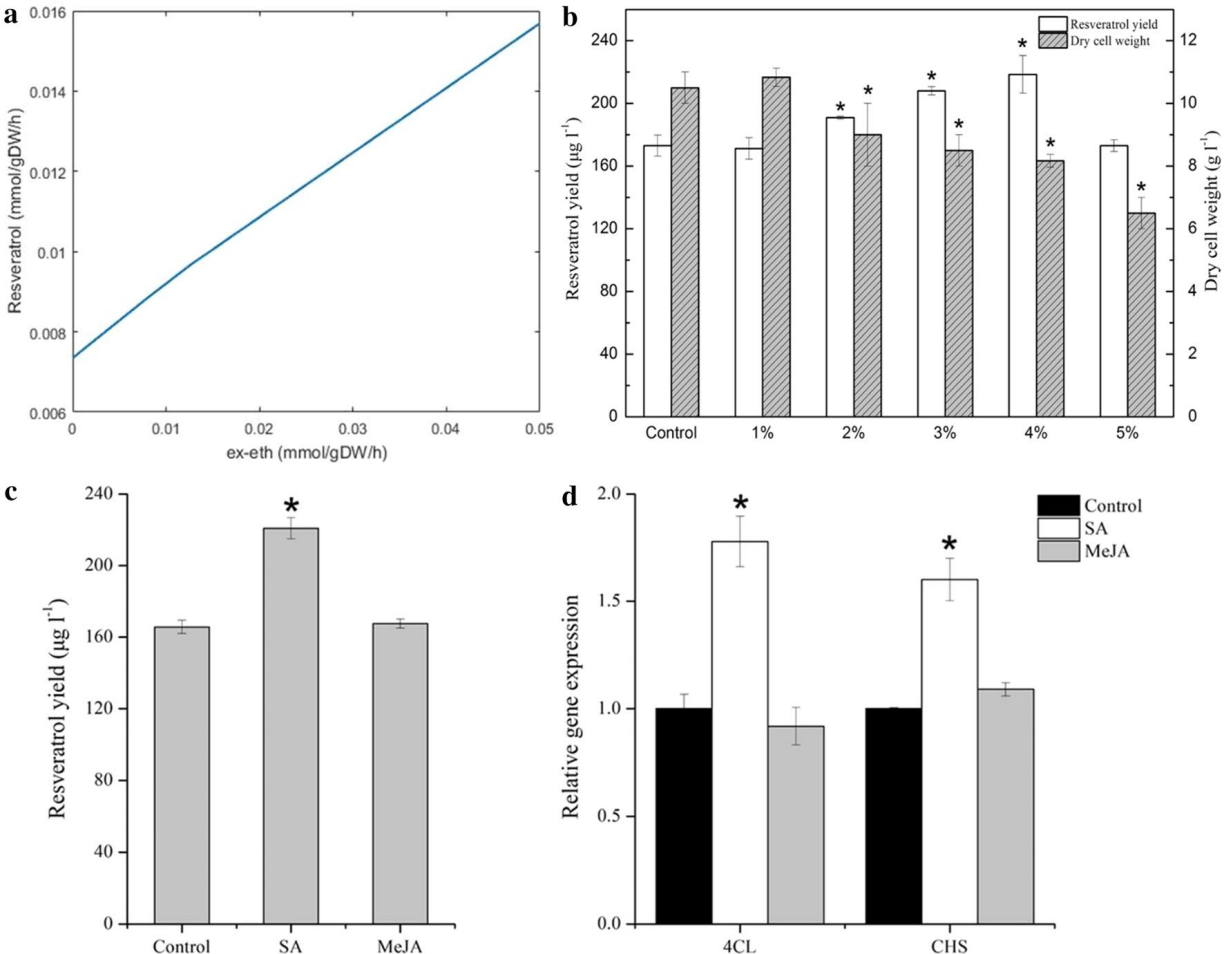
Effect of environmental or genetic disturbance on the resveratrol biosynthesis pathway. **a** Robustness analysis of the resveratrol production with ethanol uptake rate by fixing the glucose uptake rate and cell growth rate at 1.0 mmol/gDW/h and 0.055 h^−1^, respectively. **b** Effect of different concentrations of ethanol on the resveratrol production and dry cell weight. Effects of salicylic acid (SA = 100 μM) and methyl jasmonate (MeJA = 100 μM) on resveratrol production (**c**) and expression of related genes (**d**). *4CL* 4-coumaroyl-CoA synthetase, *CHS* chalcone synthase

Addition of small molecules, such as ethanol and methanol, to the medium is another efficient method to regulate the aspired production by microorganisms [1]. In addition, as an endophytic fungus of grape, *Alternaria* sp. MG1 exists in wine processing where ethanol is produced by *S. cerevisiae*. Therefore, the effect of ethanol on the resveratrol production by *Alternaria* sp. MG1 was investigated when both glucose uptake rate and cell growth rate were constrained to 1.0 mmol/gDW/h and 0.055 h^−1^, respectively. This increased the resveratrol production rate with the ethanol uptake rate that perturbed between 0 and 0.05 mmol/gDW/h (Fig. 5a). In a wet experiment, ethanol addition increased resveratrol production and inhibited the cellular growth in a concentration-dependent manner (Fig. 5b). Compared to the control, ethanol addition of 4% increased the resveratrol yield by 26.31%. However, ethanol addition of 5% caused a decrease of cell dry weight from 10.5 to 6.5 g/L with an inhibition rate of 38.10% but it did not disrupt resveratrol production. This might be because cells death happened under high ethanol conditions, causing damage or inactivity of associated enzymes.

In mechanisms, the addition of ethanol provided a new source for the generation of acetyl-CoA, accompanied by an increase of the flow of reaction g0770 (ac[c] + coa[c] + atp[c] → h[c] + accoa[c] + ppi[c] + amp[c]) from 0 to 0.038 mmol/gDW/h, thus synthesizing more malonyl-CoA (Additional file 7). Moreover, more flow of beta-d-fructose 6-phosphate produced from glycolysis channeled into the pentose phosphate pathway (PPP pathway) and led to a vast improvement of d-erythrose 4-phosphate production. This change strengthened *p*-coumaric CoA synthesis and thus generated more resveratrol.

The increase of resveratrol production by ethanol addition was also found in the fed-batch fermentation of engineered *S. cerevisiae*, where ethanol supplementation resulted in a final titer of 531.41 mg/L resveratrol, corresponding to a 27.85% increase compared to that of glucose [49]. Therefore, it can be shown that ethanol plays a dual role in the increase of resveratrol production, presumably both as a precursor and carbon source. The increase of product yield by ethanol was also found in other endophytic fungi to improve the production of camptothecin and huperzine A [50, 51].

### Identification and verification of target-genes for enhanced resveratrol production

To find the key genes related to the enhancement of resveratrol production by *Alternaria* sp. MG1, the minimization of metabolic adjustment (MOMA) algorithm was used to identify the gene candidates responsible for increased resveratrol production with the lower bound of the resveratrol exchange reaction constrained to 0.001 mmol/gDW/h. As a result, six genes were identified by computationally overexpressing all reactions with non-zero flux value in a FBA simulation (Additional file 1: Table S2). Among the proteins encoded by these six genes, UCK (EC: 2.7.1.48) participates in the nucleotide metabolism which generates UMP and ADP (an indispensable cofactor involved in the resveratrol synthesis). Overexpression of PFK (EC: 2.7.1.11) catalyzed beta-d-fructose 6-phosphate to form more beta-d-fructose 1,6-bisphosphate, which switched more flux to the PPP pathway, thus intensifying the shikimate pathway. PTAL, 4CL, CHS, and ACC are directly involved in the biosynthesis of resveratrol. Up-regulated expression of 4CL (EC: 6.2.1.12) or CHS (EC: 2.3.1.74) resulted in a twofold increase of resveratrol production in silico.

In practice, we tried to the implement salicylic acid (SA) and methyl jasmonate (MeJA) to verify whether the above identified genes were related to the enhancement of resveratrol production by *Alternaria* sp. MG1 because they had been reported as elicitors of the phenylpropane pathway genes toward accumulation of stilbenes and flavonoids. Xu et al. [52] treated *V. vinifera* cell cultures with SA and MeJA and successfully upregulated the expression of *4CL*/*CHS* and resveratrol production. In this study, 100 μM SA and MeJA were added to the PDB medium used for *Alternaria* sp. MG1 cultivation. As a result, SA induction resulted in a 33.32% increase of resveratrol production (Fig. 5c), and significantly upregulated the expression of *4CL* and *CHS* by approximately 1.8- and 1.6-fold, respectively (Fig. 5d). However, MeJA did not affect the resveratrol production nor the expression of related genes, indicating differences of metabolism regulation between plant and microorganism.

Overall, this study obtained novel results with regard to three aspects: (1) revealing the occurrence of multiple biosynthesis pathways for the production of high value secondary metabolites that were commonly found in plants; (2) constructing a genome scale of the metabolic network model (GSMM) for *Alternaria* species; (3) verifying the occurrence of the resveratrol biosynthesis pathway in a nongenetically modified microorganism at the gene level. We previously found that *Alternaria* sp. MG1 could produce resveratrol stably, although the achieved level of resveratrol production was low compared to genetically modified *E. coli* and yeast [45, 49]. With the development of technologies for modifying fungi as cell factories for the production of secondary metabolites, especially those that need complex and multiple steps for their synthesis [53, 54], *Alternaria* sp. MG1 showed potential in this field because it possesses diverse biosynthesis pathways to produce multiple metabolites with high value. The development of the gene editing tool CRISPR-Cas9 in fungal model organisms would also greatly promote the exploration of the fungal metabolism [55], such as replacing native promoters with heterologous promoters to construct an inducible pathway [56]. In addition, it is important to point out that overexpression of *CHS*/*4CL* is highly expected to lead to a significant increase in resveratrol production compared to the induction of the expression of *CHS*/*4CL* by using an elicitor indirectly. Therefore, it is reasonable to anticipate that the production of secondary metabolites could be greatly improved by using genetically modified *Alternaria* sp. MG1. Furthermore, key genes and pathways identified for the synthesis of stilbenes, flavonoids, and lignins in this study provide a rich gene resource for the production of these natural compounds using genetically engineered microorganisms. Finally, the study would also provide useful information and reference for similar studies on the exploration of other endophytic fungi.

## Conclusions

A resveratrol-producing endophytic fungus, *Alternaria* sp. MG1, was sequenced and annotated for the verification of the occurrence of phenylpropanoid—resveratrol biosynthesis pathways in a nongenetically modified microorganism at the gene level. It also details the diverse capability and key genes of *Alternaria* sp. MG1 to produce multiple phenylpropanoid metabolites, such as stilbenes, flavonoids, and lignins. In this study, a genome scale metabolic network model (GSMM) for the species *Alternaria* was constructed, providing an overall understanding of the physiological behavior and metabolic characteristics of the tested strain. Both biochemical and genetic strategies were proposed for the improvement of resveratrol production in silico and verified with wet experiments. Furthermore, important information was obtained for the exploration of key genes in this strain toward producing high value secondary metabolites. In summary, the constructed model provided a reliable platform for future studies aimed at the exploitation of other secondary metabolites in *Alternaria* sp. MG1.

## Methods

### Microorganism, medium, and cultivation

*Alternaria* sp. MG1 (code: CCTCC M 2011348), currently preserved in the China Centre for Type Culture Collection (Wuhan, China), was used throughout this study. Potato-dextrose broth (PDB), containing 200 g potato, 20 g dextrose, and 1 L distilled water, was used to cultivate the strain at 28 °C and 160 rpm for 10 days.

### Genome sequencing, assembly, and annotation

Cells at the mid-exponential stage were collected after centrifugation for 10 min at 5000×*g* and 4 °C and stored at − 80 °C until further use. Genomic DNA was extracted using the EasyPure Genomic DNA Kit (Transgene Biotech Co., Ltd., Beijing, China). For analysis, the whole-genome shotgun sequencing strategy was used and subsequent short-insert libraries (600 bp) and long-insert libraries (3 kb) were constructed using the standard protocol provided by Illumina (San Diego, USA). Paired-end sequencing was performed via the Illumina HiSeq 2500 system. Genome assembly, prediction of encoding genes, and genome annotation were conducted according to previously published methods [57–61]. The genome sequence of *V. vinifera* was downloaded from the NCBI and used as a reference to identify the resveratrol biosynthesis pathway of *Alternaria* sp. MG1.

### GC–MS analysis of stilbenoids, flavonoids, and lignins

The cells were separated from the culture by vacuum filtration. The cell-free culture was then freeze-dried into powder before use. The prepared sample was extracted with 0.5 mL extraction liquid (*V methanol*:*V chloroform* = 3:1) containing 20 μL of l-2-chlorophenylalanine (1 mg/mL stock in dH_2_O) as internal standard. The mixture was homogenized in a ball mill for 4 min at 45 Hz, then ultrasonically treated for 5 min (incubated in ice water), which was repeated twice, and finally centrifuged for 15 min at 13,000 rpm, 4 °C. The supernatant (0.4 mL) was transferred into a fresh 2 mL GC/MS glass vial and dried in a vacuum concentrator without heating. Methoxy amination hydrochloride (20 mg/mL in pyridine, 80 μL) was then added and incubated for 30 min at 80 °C. 100 μL of the BSTFA regent (1% TMCS, v/v) was added to sample aliquots, incubated for 1.5 h at 70 °C, and mixed well for GC–MS analysis. GC–TOFMS analysis was performed using an Agilent 7890 gas chromatograph system coupled with a Pegasus HT time-of-flight mass spectrometer. The system utilized a DB-5MS capillary column coated with 5% diphenyl cross-linked with 95% dimethylpolysiloxane (30 m × 250 μm inner diameter, 0.25 μm film thickness; J&W Scientific, Folsom, CA, USA). A 1 μL aliquot of the analyte was injected in splitless mode. Helium was used as carrier gas, the front inlet purge flow was 3 mL/min, and the gas flow rate through the column was 1 mL/min. The initial temperature was kept at 50 °C for 1 min, then increased to 310 °C at a rate of 10 °C/min, then kept for 8 min at 310 °C. The injection, transfer line, and ion source temperatures were 280, 270, and 220 °C, respectively. The energy was − 70 eV in electron impact mode. The mass spectrometry data were acquired in full-scan mode with the m/z range of 50–500 at a rate of 20 spectra per second after a solvent delay of 455 s. Chroma TOF 4.3X software of LECO Corporation and LECO-Fiehn Rtx5 database were used for raw peak exaction, data baselines filtering, calibration of the baseline, peak alignment, deconvolution analysis, peak identification, and integration of the peak area [62]. Metabolites were identified by matching their retention characteristics and mass fragmentation patterns with Feihn metabolomics database with a standard of the similarity value above 700. Compounds with a similarity between 200 and 700 were considered as a putative annotation.

### Identification of pterostilbene accumulation using UPLC–Qtof–MS

UPLC–MS analysis was performed using an UPLC I-Class system with a BHC C18 column (100 mm  ×  2.1 mm, 1.7 μm) coupled to VION IMS QTOF mass spectrometer (Waters Corporation, Milford, MA, USA). The mobile phase consisted of 0.1% formic acid in water (A) and acetonitrile (B) was carried with elution gradient as follows: 0 min, 5% B; 3 min, 100% B; 4 min, 100% B; 4.1 min, 5% B; 6 min, 5% B, which was delivered at 0.4 mL/min. The column and autosampler were maintained at 35 and 10 °C, respectively. The injection volume was 2 μL. The ion source was operated in positive electrospray ionization (ESI) mode under the following specific conditions: capillary voltage, 1.0 kV; source temperature, 100 °C; desolvation gas temperature, 500 °C; desolvation gas flow, 800 L/h, and cone gas flow, 50 L/h. Nitrogen (> 99.5%) was employed as desolvation and cone gas. The scan range was from 50 to 1000 m/z. The scan time for each function was set to 0.2 s. The low collision energy was set at 6 eV, and the high collision energy was ramped from 20 to 45 eV. The data were acquired and processed using the MassLynx 4.1 software (Waters Co., Milford, USA) that was incorporated with the instrument.

### Reconstruction of the metabolic network

An initial model was firstly drafted by amassing reactions from the established GSMM of related organisms *Aspergillus terreus i*JL1454 [63], *Penicillium chrysogenum i*RA1006 [64], and *Saccharomyces cerevisiae i*TO977 [65]. Reactions were collected based on orthologs between MG1 and three reference strains identified by protein sequence similarity search (identity ≥ 40%, e-value ≤ 1E−30) using BLAST [47]. Protein sequences from *A. terreus* NIH2624, *P. chrysogenum* Wisconsin 54-1255, and *S. cerevisiae* Sc288 were downloaded from UniProt [66]. Open reading frame (ORF) information for *Alternaria* sp. MG1 was uploaded to NCBI. To verify the specific metabolic capabilities, both KEGG database and literature evidences were used to assemble unique reactions that were non-existent in reference strains. Transport reactions were obtained by referring to the Transporter Classification Database (TCDB) [67]. Exchange reactions were added to the model. CELLO [68] and MetaCyc databases [69] were used to verify the subcellular localization and reaction reversibility, respectively. The obtained draft model was manually refined by removing repeated reactions, checking the mass and charge balances and filling the metabolic gaps via Cobra Toolbox 2.05 with corresponding algorithms [70].

### Biomass equation

Nucleic acids (DNA and RNA), proteins, lipids, carbohydrates, and ash were considered in the biomass equation. Total DNA and RNA were extracted [37] and their specific composition was calculated based on the genome sequence with a GC content of 50.96%. Amino acid composition was analyzed using an amino acid analyzer (L-8900, HITACHI, Japan). Lipid and cell wall compositions were assumed to be identical to that of *Alternaria alternate* [71] and *A. infectoria* [72], respectively. The cell growth and non-growth associated ATP maintenance values (GAM and NGAM, respectively) were assumed to be identical to that of *A. terreus* [63].

### Constraints-based flux analysis

Flux balance analysis (FBA) was conducted using the Cobra Toolbox 2.05 in MATLAB R2016a to analyze the flow of metabolites through a metabolic network [73]. Based on the instructions of the Cobra Toolbox, in silico analyses were performed for aspects of environmental and genetic disturbance, gene essentiality, model visualization, and robustness analysis. The optimization solvers GLPK 4.49 and Gurobi 6.5.1 were used for linear and quadratic programming [74].

### Validation of the metabolic model

To validate carbon utilization, the uptake rate of the target carbon source was set to 2.0 mmol/gDW/h, while those of other carbon sources were set to zero. The same algorithm was applied to validate the nitrogen source. The lower bounds of the exchange reactions of basic nutrient components (including glucose, H_2_O/H^+^, O_2_, NH_3_/NH_4_^+^, sulfite, and phosphate) were set as − 1000 mmol/gDW/h to mimic minimal medium in silico [74]. The biomass reaction was chosen as objective function. Quantitative validation was conducted according to reported procedures [60].

### Effect of cerulenin on the growth of MG1

In order to verify the essentialities of the genes involved in the lipid metabolism, cerulenin, a specific inhibitor of fatty acid biosynthesis was added in the medium of MG1 [45]. MG1 was inoculated onto potato dextrose agar (PDA) medium with or without 0.05 mM cerulenin. All the inoculated plates were cultivated in darkness at 28 ± 1 °C for 7 days and the colony expanding of MG1 was observed.

### Effect of ethanol disturbance on resveratrol biosynthesis

Ethanol was added in 250 mL flasks containing 100 mL PDB, to a final concentration of 1, 2, 3, 4, and 5% (v/v). To retain high biomass and enzymes activity, ethanol addition was conducted at day 4. The cells were collected from the culture after 10 days (in triplicate) to determine the cell dry weight (g/L) [75]. The resveratrol production (μg/L) in the liquid part of cell-free cultures was measured accordingly [24].

### Effect of elicitors on resveratrol biosynthesis and key gene expression

Salicylic acid and methyl jasmonate (both Sigma, Spain) were dissolved in ethanol and added to the PDB medium at a final concentration of 100 μM at day 4. Each treatment was performed in triplicate and samples without elicitor treatment were always run in parallel as control. After cultivation for 10 days, cells were collected and used for RNA extraction. Cell-free culture broth was used for the quantitative analysis of resveratrol production.

Total RNA was extracted and cDNA was synthesized via TransScript One-Step gDNA Removal and cDNA Synthesis SuperMix Kit (Transgene Biotech Co., Ltd., Beijing, China). Primers of target genes (*4CL* and *CHS*) were designed according to the transcriptome data of *Alternaria* sp. MG1 [37] and the sequences are shown in Table 4. For each gene, the expression value was normalized with respect to the reference gene *EF1* [76]. The reaction volume was set to 20 μL in accordance with the operation manual of the TransStart Tip Green qPCR SuperMix Kit (Transgene Biotech Co., Ltd., Beijing, China). All reactions were conducted in triplicate. The program and qRT-PCR analyses were performed as previously described [76, 77].

**Table 4:** Primer sequences for analysis of expression level of aimed genes

Gene	Primer sequence
*EF1*	F 5′-CACTGGTTTTGCCTTTTCCT-3′
	R 5′-TGTGGGCACCGTCAAAGT-3′
*4CL*	F 5′-GGTGGCTTGAATGTGAAT-3′
	R 5′-CAACTACTCGTCGGGAAC-3′
*CHS*	F 5′-CTCACTATCACCGCCTTCC-3′
	R 5′-CAGCACCCACGATGACG-3′

*F* forward primer, *R* reverse primer

### Accession numbers

Raw sequence data were deposited in the NCBI database under the SRA study accession numbers SRR6346758 and SRR6346759. The Whole Genome Shotgun project has been deposited at DDBJ/ENA/GenBank under the accession QPFE00000000. The version described in this paper is version QPFE01000000.

## Additional files

**Additional file 1: Figure S1.** UPLC-QTOF-MS analysis of pterostilbene accumulation in the culture of *Alternaria* sp. MG1. Extracted ion chromatogram of a sample (A) and a pterostilbene standard (B). Mass spectrum of a sample (C) and pterostilbene standard (D). The suspected pterostilbene detected in the sample (2.16 min) and pterostilbene standard (2.16 min) showed a similar retention time, molecular ion of m/z = 257.1, verifying the production of pterostilbene. **Figure S2.** Growth of *Alternaria* sp. MG1 for control (A) and cerulenin treatment (B) after cultured for 7 days on potato dextrose agar (PDA) plate and the rate of colony expanding changes with cultivation time (C). **Table S1.** Identified ATP-binding cassette (ABC) transport proteins in *Alternaria* sp. MG1. **Table S2.** Potential targets identified by MOMA which could enhance resveratrol production.

**Additional file 2.** **Dataset S1.** Metabolites detected inside cells and in the cell-free culture by setting the identification standard of similarity value greater than 200.

**Additional file 3.** **Dataset S2.** Biomass composition of *Alternaria* sp. MG1.

**Additional file 4.** **Dataset S3.** Detailed information about model *i*YL1539.

**Additional file 5.** **Dataset S4.** Result of essential genes identified by FBA using PDB medium.

**Additional file 6.** **Dataset S5.** Flux distribution with the resveratrol production rate increased from 0.0073 to 0.02 mmol/gDW/h.

**Additional file 7.** **Dataset S6.** Flux distribution involved in resveratrol synthesis with the ethanol uptake rate perturbed from 0 to 0.05 mmol/gDW/h.

## References

[1] Venugopalan A, Srivastava S (2015). Endophytes as in vitro production platforms of high value plant secondary metabolites. Biotechnol Adv.

[2] Arnold AE, Mejía LC, Kyllo D, Rojas EI, Maynard Z, Robbins N, Herre EA (2003). Fungal endophytes limit pathogen damage in a tropical tree. Proc Natl Acad Sci USA.

[3] Soliman SS, Raizada MN (2013). Interactions between co-habitating fungi elicit synthesis of taxol from an endophytic fungus in host *Taxus* plants. Front Microbiol.

[4] Kusari S, Singh S, Jayabaskaran C (2014). Rethinking production of Taxol^®^ (paclitaxel) using endophyte biotechnology. Trends Biotechnol.

[5] Kusari S, Košuth J, Čellárová E, Spiteller M (2011). Survival-strategies of endophytic *Fusarium solani* against indigenous camptothecin biosynthesis. Fungal Ecol.

[6] Shi J, Zeng Q, Liu Y, Pan Z (2012). *Alternaria* sp. MG1, a resveratrol-producing fungus: isolation, identification, and optimal cultivation conditions for resveratrol production. Appl Microbiol Biotechnol.

[7] Lu Y, Shao D, Shi J, Huang Q, Yang H, Jin M (2016). Strategies for enhancing resveratrol production and the expression of pathway enzymes. Appl Microbiol Biotechnol.

[8] Jeffery T, Ferber B (2003). One-pot palladium-catalyzed highly chemo-, regio-, and stereoselective synthesis of trans-stilbene derivatives. A concise and convenient synthesis of resveratrol. Tetrahedron Lett.

[9] Moro AV, Cardoso FSP, Correia CRD (2008). Heck arylation of styrenes with arenediazonium salts: short, efficient, and stereoselective synthesis of resveratrol, DMU-212, and analogues. Tetrahedron Lett.

[10] Charlwood BV, Rhodes MJC (1990). Secondary products from plant tissue culture.

[11] Forkmann G, Martens S (2001). Metabolic engineering and applications of flavonoids. Curr Opin Biotechnol.

[12] Monk J, Nogales J, Palsson BO (2014). Optimizing genome-scale network reconstructions. Nat Biotechnol.

[13] Feist AM, Palsson BØ (2008). The growing scope of applications of genome-scale metabolic reconstructions: the case of *E. coli*. Nat Biotechnol.

[14] Oberhardt MA, Palsson BØ, Papin JA (2009). Applications of genome-scale metabolic reconstructions. Mol Syst Biol.

[15] Ye C, Xu N, Dong C, Ye Y, Zou X, Chen X, Guo F, Liu L (2017). IMGMD: a platform for the integration and standardisation of in silico microbial genome-scale metabolic models. Sci Rep.

[16] Dicenzo GC, Checcucci A, Bazzicalupo M, Mengoni A, Viti C, Dziewit L, Finan TM, Galardini M, Fondi M (2016). Metabolic modelling reveals the specialization of secondary replicons for niche adaptation in *Sinorhizobium meliloti*. Nat Commun.

[17] Zhang H, Chao Y, Nan X, Chen C, Xiao C, Yuan F, Xu Y, Yang J, Sun D (2017). Reconstruction of a genome-scale metabolic network of *Komagataeibacter nataicola* RZS01 for cellulose production. Sci Rep.

[18] Chong J, Poutaraud A, Hugueney P (2009). Metabolism and roles of stilbenes in plants. Plant Sci.

[19] Yu CKY, Springob K, Schmidt J, Nicholson RL, Chu IK, Yip WK, Lo C (2005). A stilbene synthase gene (*SbSTS1*) is involved in host and nonhost defense responses in sorghum. Plant Physiol.

[20] Wang C, Zhi S, Liu C, Xu F, Zhao A, Wang X, Tang X, Li Z, Huang P, Yu M (2017). Isolation and characterization of a novel chalcone synthase gene family from mulberry. Plant Physiol Biochem.

[21] Yamaguchi T, Kurosaki F, Suh DY, Sankawa U, Nishioka M, Akiyama T, Shibuya M, Ebizuka Y (1999). Cross-reaction of chalcone synthase and stilbene synthase overexpressed in *Escherichia coli*. FEBS Lett.

[22] Samappito S, Page JE, Schmidt J, Deeknamkul W, Kutchan TM (2003). Aromatic and pyrone polyketides synthesized by a stilbene synthase from *Rheum tataricum*. Phytochemistry.

[23] Wenderoth M, Pinecker C, Voss B, Fischer R (2017). Establishment of CRISPR/Cas9 in *Alternaria alternata*. Fungal Genet Biol.

[24] Zhang J, Shi J, Liu Y (2013). Substrates and enzyme activities related to biotransformation of resveratrol from phenylalanine by *Alternaria* sp. MG1. Appl Microbiol Biotechnol.

[25] Sato D, Shimizu N, Shimizu Y, Akagi M, Eshita Y, Ozaki S, Nakajima N, Ishihara K, Masuoka N, Hamada H, Shimoda K, Kubota N (2014). Synthesis of glycosides of resveratrol, pterostilbene, and piceatannol, and their anti-oxidant, anti-allergic, and neuroprotective activities. Biosci Biotechnol Biochem.

[26] Rimando AM, Kalt W, Magee JB, Dewey J, Ballington JR (2004). Resveratrol, pterostilbene, and piceatannol in vaccinium berries. J Agric Food Chem.

[27] Piotrowska H, Kucinska M, Murias M (2012). Biological activity of piceatannol: leaving the shadow of resveratrol. Mutat Res Rev Mutat Res.

[28] Yang JK, Lee E, Hwang IJ, Yim D, Han J, Lee YS, Kim JH (2018). β-Lactoglobulin peptide fragments conjugated with caffeic acid displaying dual activities for tyrosinase inhibition and antioxidant effect. Bioconjug Chem.

[29] Choi J, Shin KM, Park HJ, Jung HJ, Kim HJ, Lee YS, Rew JH, Lee KT (2004). Anti-inflammatory and antinociceptive effects of sinapyl alcohol and its glucoside syringin. Planta Med.

[30] Makwana S, Choudhary R, Haddock J, Kohli P (2015). In-vitro antibacterial activity of plant based phenolic compounds for food safety and preservation. LWT Food Sci Technol.

[31] Galano A, Franciscomárquez M, Alvarezidaboy JR (2011). Mechanism and kinetics studies on the antioxidant activity of sinapinic acid. Phys Chem Chem Phys.

[32] Liu X, Wang N, Fan S, Zheng X, Yang Y, Zhu Y, Lu Y, Chen Q, Zhou H, Zheng J (2016). The citrus flavonoid naringenin confers protection in a murine endotoxaemia model through AMPK-ATF3-dependent negative regulation of the TLR4 signalling pathway. Sci Rep.

[33] Sun X, Chen RC, Yang ZH, Sun GB, Wang M, Ma XJ, Yang LJ, Sun XB (2014). Taxifolin prevents diabetic cardiomyopathy in vivo and in vitro by inhibition of oxidative stress and cell apoptosis. Food Chem Toxicol.

[34] Lambert F, Zucca J, Ness F, Aigle M (2014). Production of ferulic acid and coniferyl alcohol by conversion of eugenol using a recombinant strain of *Saccharomyces cerevisiae*. Flavour Fragr J.

[35] Zhu J, Yan L, Xu X, Zhang Y, Shi J, Jiang C, Shao D (2018). Strategies to enhance the production of pinoresinol and its glucosides by endophytic fungus (*Phomopsis* sp. XP-8) isolated from Tu-chung bark. AMB Express.

[36] Liu C, Wen X, Wu B (2016). Heterogeneous reaction of coniferyl alcohol adsorbed on silica particles with NO_3_ radicals. Atmos Pollut Res.

[37] Che J, Shi J, Gao Z, Zhang Y (2016). Transcriptome analysis reveals the genetic basis of the resveratrol biosynthesis pathway in an endophytic fungus (*Alternaria* sp. MG1) isolated from *Vitis vinifera*. Front Microbiol.

[38] Hasija S (1970). Physiological studies of *Alternaria citri* and *A. tenuis*. Mycologia.

[39] Cai Z, Kastell A, Knorr D, Smetanska I (2012). Exudation: an expanding technique for continuous production and release of secondary metabolites from plant cell suspension and hairy root cultures. Plant Cell Rep.

[40] Vos T, de la Torre Cortés P, van Gulik WM, Pronk JT, Daran-Lapujade P (2015). Growth-rate dependency of *de novo* resveratrol production in chemostat cultures of an engineered *Saccharomyces cerevisiae* strain. Microb Cell Fact.

[41] Ye C, Xu N, Chen H, Chen YQ, Chen W, Liu L (2015). Reconstruction and analysis of a genome-scale metabolic model of the oleaginous fungus *Mortierella alpina*. BMC Syst Biol.

[42] Lu H, Cao W, Ouyang L, Xia J, Huang M, Chu J, Zhuang Y, Zhang S, Noorman H (2017). Comprehensive reconstruction and in silico analysis of *Aspergillus niger* genome-scale metabolic network model that accounts for 1210 ORFs. Biotechnol Bioeng.

[43] Brzonkalik K, Hümmer D, Syldatk C, Neumann A (2012). Influence of pH and carbon to nitrogen ratio on mycotoxin production by *Alternaria alternata* in submerged cultivation. AMB Express.

[44] Juneja A, Chaplen FW, Murthy GS (2016). Genome scale metabolic reconstruction of *Chlorella variabilis* for exploring its metabolic potential for biofuels. Bioresour Technol.

[45] Lim CG, Fowler ZL, Hueller T, Schaffer S, Koffas MA (2011). High-yield resveratrol production in engineered *Escherichia coli*. Appl Environ Microbiol.

[46] Che J, Shi J, Gao Z, Zhang Y (2016). A new approach to produce resveratrol by enzymatic bioconversion. J Microbiol Biotechnol.

[47] Mishra P, Park GY, Lakshmanan M, Lee HS, Lee H, Chang MW, Ching CB, Ahn J, Lee DY (2016). Genome-scale metabolic modeling and in silico analysis of lipid accumulating yeast *Candida tropicalis* for dicarboxylic acid production. Biotechnol Bioeng.

[48] Xu N, Ye C, Chen X, Liu J, Liu L (2017). Genome-scale metabolic modelling common cofactors metabolism in microorganisms. J Biotechnol.

[49] Li M, Kildegaard KR, Chen Y, Rodriguez A, Borodina I, Nielsen J (2015). *De novo* production of resveratrol from glucose or ethanol by engineered *Saccharomyces cerevisiae*. Metab Eng.

[50] Venugopalan A, Srivastava S (2015). Enhanced camptothecin production by ethanol addition in the suspension culture of the endophyte, *Fusarium solani*. Bioresour Technol.

[51] Zhao XM, Wang ZQ, Shu SH, Wang WJ, Xu HJ, Ahn YJ, Wang M, Hu X (2013). Ethanol and methanol can improve huperzine A production from endophytic *Colletotrichum gloeosporioides* ES026. PLoS ONE.

[52] Xu A, Zhan JC, Huang WD (2015). Effects of ultraviolet C, methyl jasmonate and salicylic acid, alone or in combination, on stilbene biosynthesis in cell suspension cultures of *Vitis vinifera* L. cv. Cabernet Sauvignon. Plant Cell Tissue Organ Cult.

[53] Nevalainen KH, Te’o VS, Bergquist PL (2005). Heterologous protein expression in filamentous fungi. Trends Biotechnol.

[54] Nielsen JC, Nielsen J (2017). Development of fungal cell factories for the production of secondary metabolites: linking genomics and metabolism. Synth Syst Biotechnol.

[55] Blazeck J, Alper H (2010). Systems metabolic engineering: genome-scale models and beyond. Biotechnol J.

[56] Kennedy J, Turner G (1996). δ-(l-α-Aminoadipyl)-l-cysteinyl-d-valine synthetase is a rate limiting enzyme for penicillin production in *Aspergillus nidulans*. Mol Gen Genet.

[57] Tarailo-Graovac M, Chen N (2009). Using RepeatMasker to identify repetitive elements in genomic sequences. Curr Protoc Bioinform.

[58] Wicker T, Oberhaensli S, Parlange F, Buchmann JP, Shatalina M, Roffler S, Ben-David R, Doležel J, Šimková H, Schulze-Lefert P (2013). The wheat powdery mildew genome shows the unique evolution of an obligate biotroph. Nat Genet.

[59] Xu N, Ye C, Chen X, Liu J, Liu L, Chen J (2016). Genome sequencing of the pyruvate-producing strain *Candida glabrata* CCTCC M202019 and genomic comparison with strain CBS138. Sci Rep.

[60] Ye C, Qiao W, Yu X, Ji X, Huang H, Collier JL, Liu L (2015). Reconstruction and analysis of the genome-scale metabolic model of *Schizochytrium limacinum* SR21 for docosahexaenoic acid production. BMC Genom.

[61] Zerbino DR, Birney E (2008). Velvet: algorithms for *de novo* short read assembly using de Bruijn graphs. Genome Res.

[62] Kind T, Wohlgemuth G, Lee DY, Lu Y, Palazoglu M, Shahbaz S, Fiehn O (2009). FiehnLib: mass spectral and retention index libraries for metabolomics based on quadrupole and time-of-flight gas chromatography/mass spectrometry. Anal Chem.

[63] Liu J, Gao Q, Xu N, Liu L (2013). Genome-scale reconstruction and in silico analysis of *Aspergillus terreus* metabolism. Mol BioSyst.

[64] Agren R, Liu L, Shoaie S, Vongsangnak W, Nookaew I, Nielsen J (2013). The RAVEN toolbox and its use for generating a genome-scale metabolic model for *Penicillium chrysogenum*. PLoS Comput Biol.

[65] Österlund T, Nookaew I, Bordel S, Nielsen J (2013). Mapping condition-dependent regulation of metabolism in yeast through genome-scale modeling. BMC Syst Biol.

[66] Apweiler R, Bairoch A, Wu CH, Barker WC, Boeckmann B, Ferro S, Gasteiger E, Huang H, Lopez R, Magrane M, Martin MJ, Natale DA, O’Donovan C, Redaschi N, Yeh LS (2004). UniProt: the universal protein knowledgebase. Nucleic Acids Res.

[67] Saier MH, Tran CV, Barabote RD (2006). TCDB: the transporter classification database for membrane transport protein analyses and information. Nucleic Acids Res.

[68] Yu CS, Chen YC, Lu CH, Hwang JK (2006). Prediction of protein subcellular localization. Proteins.

[69] Caspi R, Altman T, Billington R, Dreher K, Foerster H, Fulcher CA, Holland TA, Keseler IM, Kothari A, Kubo A (2013). The MetaCyc database of metabolic pathways and enzymes and the BioCyc collection of pathway/genome databases. Nucleic Acids Res.

[70] Wang Y, Xu N, Ye C, Liu L, Shi Z, Wu J (2015). Reconstruction and in silico analysis of an *Actinoplanes* sp. SE50/110 genome-scale metabolic model for acarbose production. Front Microbiol.

[71] Häggblom P, Unestam T (1979). Blue light inhibits mycotoxin production and increases total lipids and pigmentation in *Alternaria alternata*. Appl Environ Microbiol.

[72] Fernandes C, Anjos J, Walker LA, Silva BM, Cortes L, Mota M, Munro CA, Gow NA, Gonçalves T (2014). Modulation of *Alternaria infectoria* cell wall chitin and glucan synthesis by cell wall synthase inhibitors. Antimicrob Agents Chemother.

[73] O’Brien EJ, Monk JM, Palsson BO (2015). Using genome-scale models to predict biological capabilities. Cell.

[74] Fondi M, Maida I, Perrin E, Mellera A, Mocali S, Parrilli E, Tutino ML, Liò P, Fani R (2015). Genome-scale metabolic reconstruction and constraint-based modelling of the Antarctic bacterium *Pseudoalteromonas haloplanktis* TAC125. Environ Microbiol.

[75] Hefnawy MA, Gharieb MM, Shaaban MT, Soliman AM (2017). Optimization of culture condition for enhanced decolorization of direct blue dye by *Aspergillus flavus* and *Penicillium canescens*. J Appl Pharm Sci.

[76] Che J, Shi J, Lu Y, Liu Y (2016). Validation of reference genes for normalization of gene expression by qRT-PCR in a resveratrol-producing entophytic fungus (*Alternaria* sp. MG1). AMB Express.

[77] Livak KJ, Schmittgen TD (2001). Analysis of relative gene expression data using real-time quantitative PCR and the 2^−ΔΔCT^ method. Methods.

[78] Côté CD, Rasmussen BA, Duca FA, Zadehtahmasebi M, Baur JA, Daljeet M, Breen DM, Filippi BM, Lam TK (2015). Resveratrol activates duodenal Sirt1 to reverse insulin resistance in rats through a neuronal network. Nat Med.

[79] Taniguchi T, Iizumi Y, Watanabe M, Masuda M, Morita M, Aono Y, Toriyama S, Oishi M, Goi W, Sakai T (2016). Resveratrol directly targets DDX5 resulting in suppression of the mTORC1 pathway in prostate cancer. Cell Death Dis.

[80] Li Y, Yang P, Chang Q, Wang J, Liu J, Lv Y, Wang T, Gao B, Zhang Y, Yu LL (2017). Inhibitory effect of piceatannol on TNF-α mediated inflammation and insulin resistance in 3T3-L1 adipocytes. J Agric Food Chem.

[81] Yasir F, Wahab A, Choudhary MI (2017). Protective effect of dietary polyphenol caffeic acid on ethylene glycol-induced kidney stones in rats. Urolithiasis.

[82] Matejczyk M, Świsłocka R, Golonko A, Lewandowski W, Hawrylik E (2018). Cytotoxic, genotoxic and antimicrobial activity of caffeic and rosmarinic acids and their lithium, sodium and potassium salts as potential anticancer compounds. Adv Med Sci.

[83] Silambarasan T, Manivannan J, Priya MK, Suganya N, Chatterjee S, Raja B (2014). Sinapic acid prevents hypertension and cardiovascular remodeling in pharmacological model of nitric oxide inhibited rats. PLoS ONE.

[84] Zhang N, Hu Z, Zhang Z, Liu G, Wang Y, Ren Y, Wu X, Geng F (2017). Protective role of naringenin against AÎ^2^25-35-caused damage via ER and PI3K/Akt-mediated pathways. Cell Mol Neurobiol.

[85] Wang Y, Wang Q, Bao X, Ding Y, Shentu J, Cui W, Chen X, Wei X, Xu S (2018). Taxifolin prevents β-amyloid-induced impairments of synaptic formation and deficits of memory via the inhibition of cytosolic phospholipase A2/prostaglandin E2 content. Metab Brain Dis.

[86] Sankar NR, Sreeramulu A (2009). Effect of carbon and nitrogen sources on growth, bio-mass production and antifungal metabolites by *Alternaria alternata* and *Cladosporium oxysporum*. J Plant Dis Sci.

[87] Brian PW, Curtis PJ, Hemming HG, Jefferys EG, Unwin CH, Wright JM (1951). Alternaric acid; a biologically active metabolic product of *Alternaria solani* (Ell. & Mart.) Jones & Grout; its production, isolation and antifungal properties. Microbiology.

[88] Isshiki A, Akimitsu K, Yamamoto M, Yamamoto H (2001). Endopolygalacturonase is essential for citrus black rot caused by *Alternaria citri* but not brown spot caused by *Alternaria alternata*. Mol Plant Microbe Interact.

[89] Saad S, Hagedorn DJ (1970). Growth and nutrition of an *Alternaria* pathogenic to snapbeans. Pathology.

