# Supplementary material for: Genomic sequencing, genome-scale metabolic network reconstruction, and in silico flux analysis of the grape endophytic fungus Alternaria sp. MG1
Source: Microb Cell Fact. 2019 Jan 24;18:13. doi: 10.1186/s12934-019-1063-7 (PMC6345013; doi:10.1186/s12934-019-1063-7)
Supplement: Supplementary file 1 — Additional file 1: Figure S1. UPLC-QTOF-MS analysis of pterostilbene accumulation in the culture of Alternaria sp. MG1. Extracted ion chromatogram of a sample (A) and a pterostilbene standard (B). Mass spectrum of a sample (C) and pterostilbene standard (D). The suspected pterostilbene detected in the sample (2.16 min) and pterostilbene standard (2.16 min) showed a similar retention time, molecular ion of m/z = 257.1, verifying the production of pterostilbene. Figure S2. Growth of Alternaria sp. MG1 for control (A) and cerulenin treatment (B) after cultured for 7 days on potato dextrose agar (PDA) plate and the rate of colony expanding changes with cultivation time (C). Table S1. Identified ATP-binding cassette (ABC) transport proteins in Alternaria sp. MG1. Table S2. Potential targets identified by MOMA which could enhance resveratrol production. [file 12934_2019_1063_MOESM1_ESM.pdf]

Additional file 1

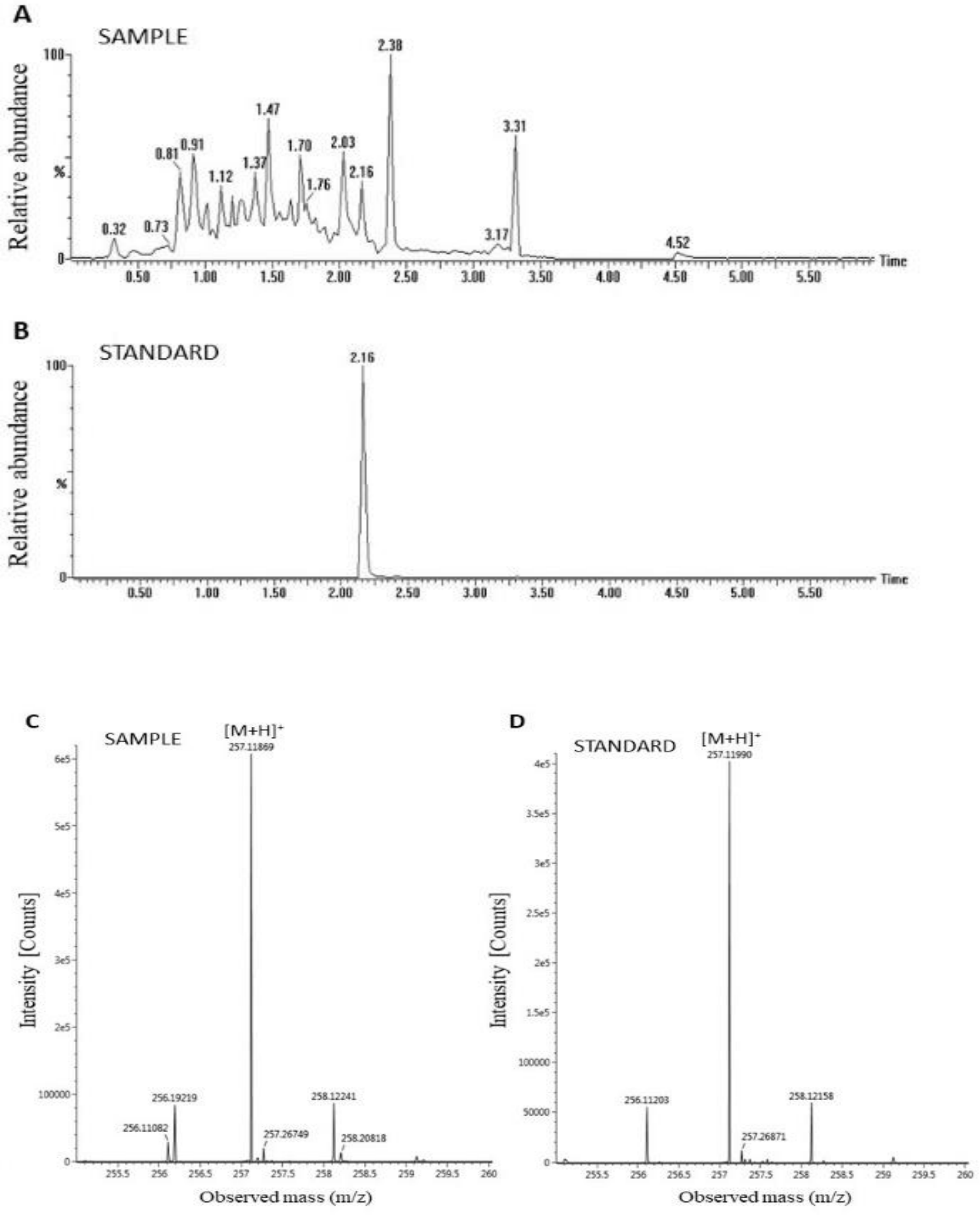

Figure S1 UPLC-QTOF-MS analysis of pterostilbene accumulation in the culture of *Alternaria* sp. MG1. Extracted ion chromatogram of a sample (A) and a pterostilbene standard (B). Mass spectrum of a sample (C) and pterostilbene standard (D). The

suspected pterostilbene detected in the sample (2.16 min) and pterostilbene standard (2.16 min) showed a similar retention time, molecular ion of m/z = 257.1, verifying the production of pterostilbene.

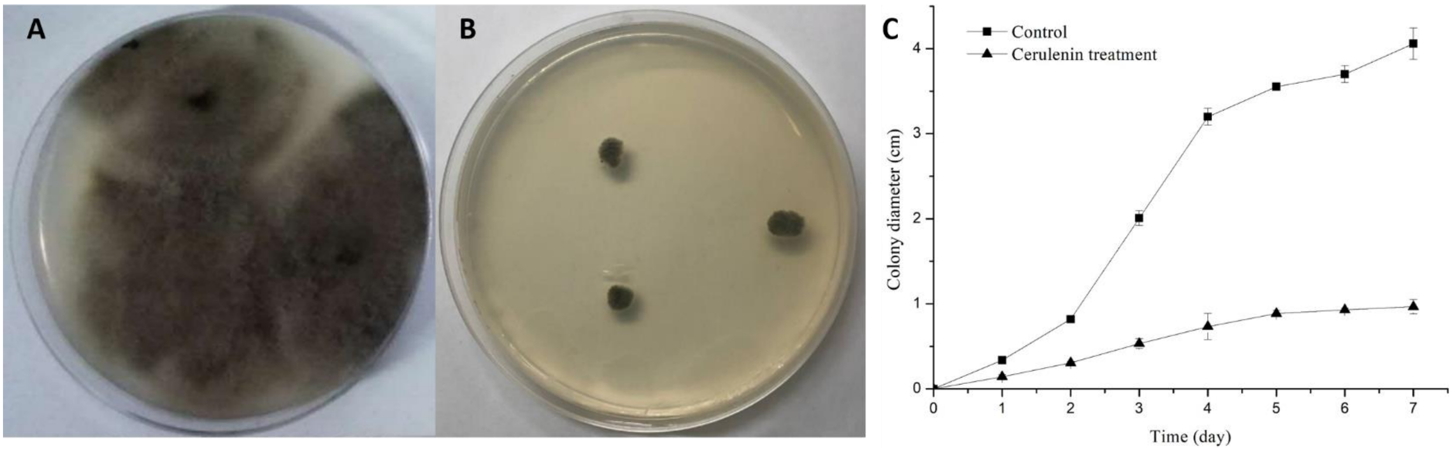

Figure S2 Growth of *Alternaria* sp. MG1 for control (A) and cerulenin treatment (B) after cultured for 7 days on potato dextrose agar (PDA) plate and the rate of colony expanding changes with cultivation time (C).

Table S1: Identified ATP-binding cassette (ABC) transport proteins in *Alternaria* sp. MG1

Gene	Cellular localization	Identity value
Gglean013490.1	M	55.02
Gglean000215.1	P	43.56
Gglean000807.1	P	69.40
Gglean001025.1	P	49.34
Gglean001838.1	P	49.76
Gglean003169.1	P	45.71
Gglean003936.1	P	44.92
Gglean005101.1	P	49.80
Gglean005614.1	P	44.63
Gglean006111.1	P	43.63
Gglean006385.1	P	41.08
Gglean006420.1	P	43.44
Gglean006857.1	P	46.31
Gglean007220.1	P	51.95
Gglean007738.1	P	48.59
Gglean008199.1	M	40.30
Gglean009837.1	P	47.56
Gglean010371.1	P	45.99
Gglean011424.1	P	50.00
Gglean011631.1	P	45.99
Gglean012071.1	P	44.21
Gglean012237.1	P	64.53
Gglean012433.1	P	45.76

M: Mitochondrial; P: Plasma membrane

Table S2: Potential targets identified by MOMA which could enhance resveratrol production

Reaction	EC number	Enzyme name	VbOE	VresOE	VbWIDE	VresWIDE	fPH
g0182	6.4.1.2	acetyl-CoA carboxylase (AAC)	0.0457	0.0097	0.0566	0.001	7.83
g0270	2.7.1.48	uridine kinase (UCK)	0.0346	0.0036	0.0566	0.001	2.20
g2173	2.3.1.74	chalcone synthase (CHS)	0.0562	0.002	0.0566	0.001	1.99
g0852	2.7.1.11	6-phosphofructokinase (PFK)	0.0407	0.0046	0.0566	0.001	3.31
g2185	4.3.1.25	phenylalanine/tyrosine ammonia-lyase (PTAL)	0.0562	0.002	0.0566	0.001	1.99
g2188	6.2.1.12	4-coumaroyl-CoA synthetase (4CL)	0.0561	0.002	0.0566	0.001	1.98

Vb: growth rate; Vres: resveratrol synthesis rate; OE: overexpressed strain; WIDE: wild type
